# Stress is not so bad—cortisol level and psychological functioning after 8-week HIIT program during pregnancy: a randomized controlled trial

**DOI:** 10.3389/fpubh.2023.1307998

**Published:** 2024-01-08

**Authors:** Dominika Wilczyńska, Tamara Walczak-Kozłowska, Rita Santos-Rocha, Radosław Laskowski, Anna Szumilewicz

**Affiliations:** ^1^Department of Physical Culture, Gdańsk University of Physical Education and Sport, Gdańsk, Poland; ^2^Department of Neuropsychology, Institute of Psychology, University of Gdańsk, Gdańsk, Poland; ^3^ESDRM Department of Physical Activity and Health, Sport Sciences School of Rio Maior, Polytechnic Institute of Santarém, Santarém, Portugal; ^4^CIPER Interdisciplinary Centre for the Study of Human Performance, Faculty of Human Kinetics (FMH), University of Lisbon, Lisbon, Portugal

**Keywords:** pregnancy, hair cortisol level, exercise, high intensity interval training, mental health, fear of childbirth

## Abstract

**Background:**

Amid extensive pregnancy exercise research, the impact of High Intensity Interval Training (HIIT) on pregnant women’s mental health is underexplored. Despite exercise benefits, it can trigger stress responses like elevated cortisol. This study fills the gap by investigating correlations between hair cortisol levels, mental health, and HIIT effects in pregnant women.

**Methods:**

We conducted a randomized control trial among 38 Caucasian women in uncomplicated, singleton pregnancy (age 31.11 ± 4.03 years, 21.82 ± 4.30 week of gestation; mean ± SD). The experimental group comprised 22 women engaged in an 8-week high-intensity interval training program (HIIT). The comparative group consisted of 16 pregnant women undergoing an 8-week educational program (EDU). Before and after the interventions, all women were evaluated using the following tools: Hair cortisol level measurements, Beck Depression Inventory – II for depressive symptoms assessment, Childbirth Attitudes Questionnaire for childbirth fear measurement, 12-item Short Form Health Survey to gage health-related quality of life, International Physical Activity Questionnaire for physical activity level estimation, and a Progressive maximal exercise test to evaluate maternal exercise capacity.

**Results:**

The key finding of our study reveals that women engaged in the HIIT intervention exhibited a distinct cortisol production pattern in contrast to the EDU group practicing standard moderate intensity physical activity. In the HIIT group, there was an increase in hair cortisol levels, while the EDU group showed a notable decrease. Remarkably, HIIT stimulated cortisol production without adversely impacting fear of childbirth and psychophysical condition during pregnancy. In fact, only the HIIT group showed a significant enhancement in mental health.

**Conclusion:**

No links were discovered between hair cortisol levels and the severity of depressive symptoms, psychophysical well-being, or fear of childbirth. Hence, based on our research, employing cortisol levels during pregnancy as an indicator of negative stress or depression risk appears unwarranted.

## Introduction

Pregnancy is a transformative period in a woman’s life, marked by various physiological and psychological changes. The overall well-being of pregnant women is not only influenced by physical health but also by their mental health status. Pregnancy can bring about such emotional challenges as increased levels of stress, anxiety, and depression, as well as concerns about childbirth (1–3). Maintaining good mental health during pregnancy is crucial for the well-being of both the mother and the unborn child (4). Numerous studies have demonstrated the positive effects of exercise on mental health outcomes, including reducing symptoms of depression and anxiety, improving mood, and enhancing overall psychological well-being in various populations (5–8). As a result, official guidelines on physical activity during pregnancy published by credible organization representing the health or sport medicine sectors contain recommendations for regular physical activity as a form of prevention of pre-and postnatal depression and anxiety (9).

According to Guszkowska (10), supervised exercise during pregnancy apart from prevention and reduction of prenatal depression and depressive symptoms (11, 12) may alleviate the fear of childbirth. However, it is important to note that exercise itself, despite its numerous health advantages, can increase the body’s stress response (13). Nonetheless, individual responses to stress and the mindset toward stress can vary significantly among individuals. What is “negatively stressful” for some people may be “positively stressful” for others depending on mental and environmental resources (14). Therefore, psychological theory suggests that stress is not inherently maladaptive, although traditional assumptions tend to conceptualize stress as inherently dysfunctional. Noteworthy models such as the holistic stress model of Nelson and Simmons, and the transactional approach of Lazarus and Folkman also emphasize that stress can be both positive and negative (15, 16). This study, using partial consensus, defines stress as the subjective response to stressors which can be (a) distress, the adverse, undesired, and harmful response to stressors, and (b) eustress, the favorable, desirable, and beneficial response to stressors, depending on how the stressors are perceived by the individual. Viewed as distinct constructs rather than as extremes on a continuum, individuals can experience both distress and eustress simultaneously (17).

Maternal prenatal stress is commonly examined by focusing on two different components, psychological stress and biological correlates of stress. Psychological stress is assessed through stressful life events, aspects of psychological symptomatology, anxiety and depression or by assessing subjectively perceived stress. On the other hand, cortisol is studied as biological correlate of stress in pregnant respondents (18–20). Pregnancy-related worries and stress activate the hypothalamic–pituitary–adrenal axis (HPA), triggering cortisol release that may affects fetal development (21). Research on antenatal care in Europe revealed that 24% of pregnant women experienced anxiety, and 22% faced depression during the second and third trimesters (22). In contrast, United States reported 10% of pregnant women experiencing anxiety (23), which positively correlates with cortisol (24). A systematic review on hair cortisol during pregnancy reports that the majority of pregnant respondents fall within the range of 0 to 34.15 pg./mg during trimesters 1 and 2, and between 8.59 and 44 pg./mg in trimester 3. However, notably wide ranges, exemplified by values exceeding 250 pg./mg, and markedly elevated values, reaching averages in the 200 s to 300 s and peaking at 768 pg./mg, are observed specifically from one laboratory. The authors underline that establishing a reference range for hair cortisol concentrations throughout pregnancy proves challenging due to acknowledged factors like variations in values obtained from different laboratories and assay types (25). Stress response in pregnant women can be estimated by cortisol level in hair samples which is non-invasive method. Traditional methods of cortisol assessment, such as blood or saliva sampling, provide insights into acute stress levels. However, they fail to capture long-term stress experiences. In recent years, hair cortisol analysis has emerged as a promising technique to assess chronic stress. Hair strands provide a time-averaged measure of cortisol levels over extended periods, offering a unique opportunity to investigate stress experiences over several weeks or months (26). Nevertheless, Budnik-Przybylska et al. (27), analyzing self-reported psychosocial and physiological stress in pregnant and non-pregnant women, found no significant association between hair cortisol levels and perceived stress. These results challenge previous findings and confirm that physiological stress is not always a determinant of psychological distress.

The impact of stress on physical and mental health has long been acknowledged. Excessive and prolonged exposure to distress can have detrimental effects, contributing to the development of various mental health disorders, cardiovascular diseases, and impaired immune function. Conversely, eustress, when appropriately managed, can enhance cognitive and behavioral functioning (14), engagement in exercising (28), resilience and overall well-being (29). Moreover, responding to demanding stressors is theorized to differentially impact psychological, behavioral, and physical health. This demanding stressor could be high-intensity, anaerobic physical activity (13). Although research on the effects of exercise during pregnancy is extensive, the specific impact of High Intensity Interval Training (HIIT) on mental health outcomes in pregnant women remains relatively unexplored.

HIIT consists of brief bouts of activity (e.g., 20–30 s workout intervals) followed by short recovery periods and can include a considerable range of exercise durations and intensities (30). Either way, without a doubt these bouts of intense exercise induce an important physiological stress response that can be observed in various body systems (endocrine, muscular, respiratory, cardiovascular). More precisely, the hypothalamic–pituitary–adrenal (HPA) axis responds to stress by causing the secretion of corticotropin-releasing hormone (CRH) from the hypothalamus which stimulates the pituitary gland to secrete adrenocorticotropin hormone (ACTH). This pituitary hormone regulates adrenal function and thus the release of cortisol in response to stress (31). This favors catabolic over anabolic processes, which is beneficial for organism adaptation in the short term (32). Literature confirms that cortisol levels surge in reaction to acute physical activity. However, it is important to note that this occurs only when appropriate intensity thresholds have been achieved. The literature is unclear on the minimum exercise intensity required to trigger a cortisol response, with conflicting findings around the commonly suggested 60% of VO2max threshold (33). Is worth to mention that 40–60% of VO_2max_ is defined as moderate-intensity physical activity while 60–85% of VO_2max_ is described as vigorous-intensity of physical activity (34). Moreover, the elevation of cortisol levels in response to exercise is influenced not only by intensity but also by duration, or a combination of both. Therefore, when investigating the cortisol response, it is essential to maintain a consistent level of either exercise duration or intensity while adjusting the other to distinctly discern the individual impact of each contributing factor (33). The studies on female participants response to prolonged exercise intervention of low or moderate-intensity (yoga, aerobic outdoor/indoor exercises) showed no changes in cortisol or slight increase pre-intervention which moved to a more regular cortisol level post-intervention (35, 36). Though aerobic exercise is distinguished by low to moderate intensity and high volume, HIIT involves high intensity and short duration, leading to potentially distinct cortisol responses. A review and meta-analysis conducted by Dote-Montero et al. (37) revealed that cortisol levels experience an immediate increase after a single HIIT session, subsequently dropping below baseline levels, and ultimately returning to baseline values after 24 h.

This study is a part of the HIIT Mama project and continuation of published study, Wilczyńska et al. (8). This time we are filling the knowledge gap by exploring the relationship between hair cortisol levels, mental health outcomes, and the fitness effects of HIIT in pregnant women. Therefore, our objective was to address the following study questions: what is the impact of HIIT on cortisol levels, and are hair cortisol levels associated with symptoms of depression, fear of childbirth, mental and physical health, as well as exercise capacity in pregnant women?

## Methods

**Figure 1:**
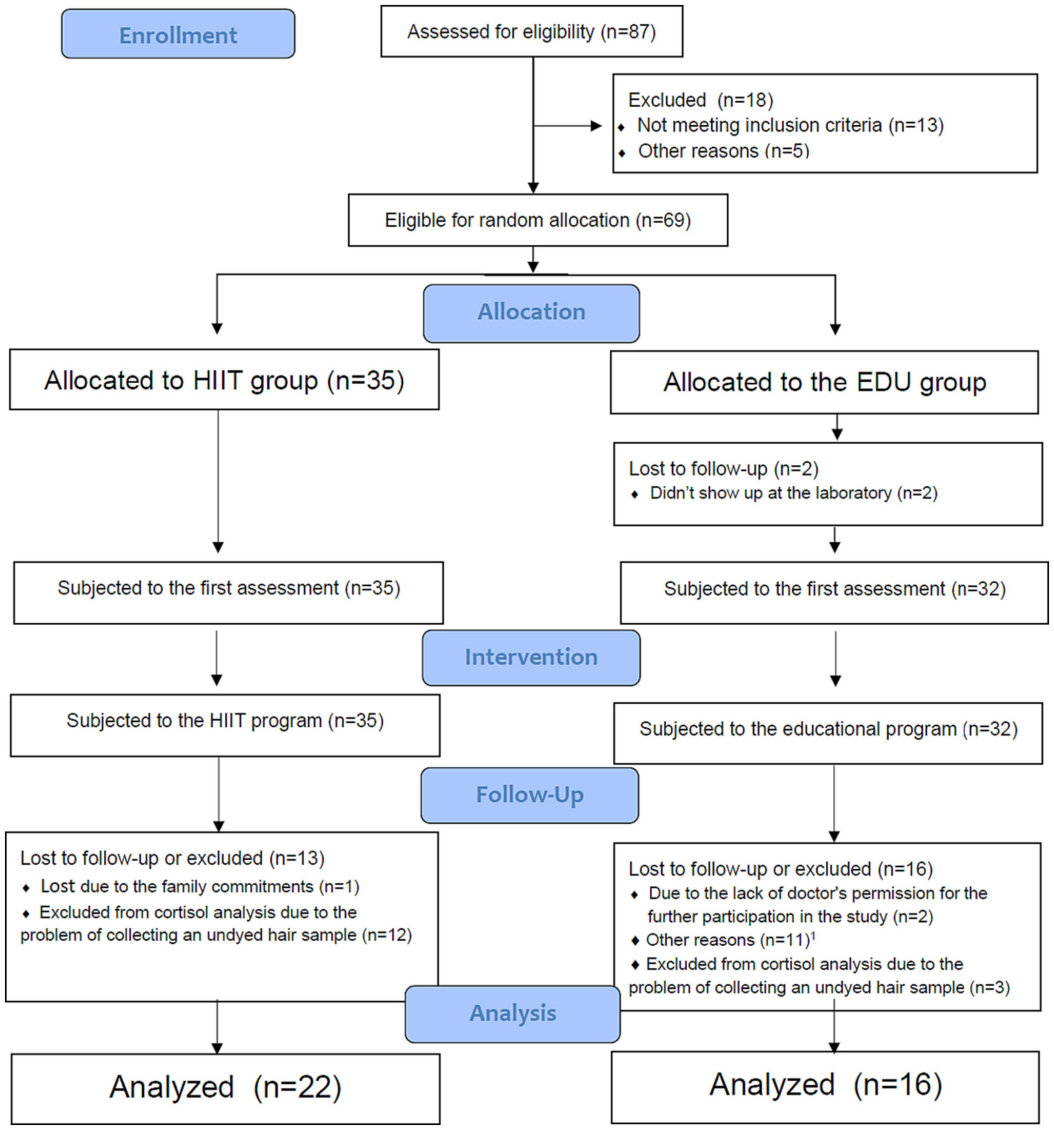
Flow of participants through the study. HIIT, high intensity interval training; EDU, educational. ^1^Other reasons: no interest to continue the program (*n* = 6); preterm birth (*n* = 1); taking medications which could influence the outcomes of the study (*n* = 1); not feeling well on the day of the second assessment (*n* = 2); did not provide the reason (*n* = 2).

A group of 69 Caucasian women in uncomplicated, singleton pregnancy who voluntarily responded to our mass media invitation were eligible to participate in the study. To randomly allocate the participants into the high intensity interval training group (HIIT group) or educational intervention (EDU group) and to avoid the “contamination effect,” feasibility and ethical issues during the study implementation we used the pipeline arm-focused randomization (PAFR) model, based on the assumptions of pipeline randomization (38) or stepped wedge randomization (39). The allocation ratio was 1:1. The flow of the participants through the study is presented in Figure 1. Twenty-six did not complete the 8-week interventions with pre-and post-intervention assessments. Fifteen participants were excluded due to the impossibility of evaluating cortisol levels in the hair due to hair dyeing in a period too short from the date of sample collection. In sum, the HIIT group consisted of 22 women (age 30 ± 4 years, 21 ± 4 week of gestation; mean ± SD) who participated in an 8-week high intensity interval training program. The EDU group was constituted of 16 pregnant women (age 32 ± 4 years, 23 ± 4 week of gestation; mean ± SD) who attended 8-week educational program on a healthy lifestyle and physical activity in the perinatal period. The eligibility criterion was a course of pregnancy allowing participation in physical activities adapted to pregnant women, confirmed by the routine obstetric consultation. Among the exclusion criteria there were contraindications to increased physical effort or other conditions that, according to the researchers, could threaten the health or safety of the participants or could significantly affect the quality of the collected data.

### Hair cortisol level measurements

Hair strands were taken from the scalp near the posterior vertex region of each participant. Cortisol was assessed of the 1-cm segments. The procedure of hair segment analysis followed a modified version of the laboratory protocol previously described in Budnik-Przybylska et al. (27). Each hair segment was washed twice with 4 mL isopropanol for 3 min to remove external contaminants from the outer hair. The hair was then placed in a tissue paper and dried for 12 h. Next, the hair was powdered in liquid nitrogen, and the powder was poured into the samples. Then we heated it at 50°C for 2 h. For steroid extraction, 1 mL of methanol was added; then we heated it at 50°C for 16 h. Following steroid extraction, the samples were spun in a microcentrifuge (7,000 rpm for 30 s), and 1 mL of the clear supernatant was transferred into a new vial. The methanol was evaporated under a constant stream of nitrogen at 55°C until the samples were completely dried. Finally, 0.4 mL phosphate buffer was added, and the vials were vortexed for 15 s. We determined cortisol using the DetectX, Cortisol Enzyme immunoassay kit (Arbor Assays, MI, 48108–3,284 United States) and the ELISA method (27, 40). The same procedure was repeated before and after 8-week intervention (27).

### Beck depression inventory—II (BDI-II)

We used BDI-II (Beck Depression Inventory—II) in order to measure the occurrence and severity of depression symptoms. This tool is a patient-rated 21-item inventory and participants are required to rate on a Likert scale from 0 to 3 the severity of the depressive symptoms occurring in the last 2 weeks. The obtained scores can range from 0 to 63. The BDI-II has the following clinical cutoff points: 0–13: no depression; 14–19: mild depression; 20–28: moderate depression; 29–63: severe depression. The BDI-II has established psychometric properties (41). In our study, the Cronbach alpha for the HIIT group and EDU group in BDI-II assessment was 0.78 and 0.73, respectively.

### Fear of childbirth

The Childbirth Attitudes Questionnaire (CAQ) (42), was used to measure fear of childbirth (43). This tool is a 16-item questionnaire, with a 4-point Likert scale. The total score is ranging from 16 to 64, and higher scores indicate higher severity of the fear of childbirth. The Cronbach alpha for both HIIT group and EDU group was 0.90.

### 12-item short form health survey (SF-12)

Health-related quality of life was measured with the 12-item Short Form Health Survey (SF-12) questionnaire which consists of a physical (PCS) and a mental (MCS) subscales (44). It is a self-administered questionnaire, which measures physical and mental health status. Responses to questions in this questionnaire are dichotomous (yes/no), ordinal (excellent to poor), or expressed by a frequency (always to never). Obtained scores allow the calculation of Physical Component Summary (PCS) and Mental Component Summary (MCS) scores. In this tool the higher the score, the better the health status. The SF-12 reliability from the study of Ware et al., is 0.93 (Cronbach alpha) (44). In our study, the Cronbach alpha for HIIT group and EDU group was 0.75 and 0.67, respectively.

### International physical activity questionnaire

The short form of International Physical Activity Questionnaire was used to measure the level of physical activity (45). This tool has shown acceptable measurement properties. It provides information on weekly PA levels in multiples of the resting metabolic rate (METs).

### Progressive maximal exercise test

To assess maternal exercise capacity, we determined the oxygen consumption during a progressive maximal test on a cycloergometer with electronically regulated load (Viasprint 150P) and respiratory gas analyzer (Oxycon Pro, Erich JAEGER GmbH, Germany). For the details of the test protocol see our previous study (46). In order to measure the maximal oxygen capacity (VO_2max_) we used the highest value of oxygen uptake, which was maintained for 15 s. The anaerobic threshold (AT) values, such as oxygen uptake at AT (VO_2_/AT) and heart rate at AT (HR/AT) were established using the V-slope method (47).

### Experimental training and educational interventions

The HIIT intervention consisted of three 60-min training sessions a week to which women participated for 8 weeks. Participants started with the warm-up combined with short educational guide on how to perform exercises in the main part (this part of the session lasted approx. 7–10 min). The main part lasted approx. 15–20 min and was performed in the form of high intensity intervals. The individual heart rate at anaerobic threshold (HR/AT) was determined for each woman on the basis of the progressive maximal exercise test. The HR/AT was set, on average, at 88 ± 5% of maximal heart rate. With the use of a heart rate monitor (Polar RS400, Finland) women were supposed to exceed the value of HR/AT in workout intervals for as long as they felt comfortable. Additionally, we monitored the exercise intensity with the use of the 0–10 Borg Rating of Perceived Exertion (RPE) and the Talk Test (48, 49).

The workout intervals included exercises that involved the main muscle groups (e.g., squats, lunges, jumps, combined with the upper body movements). They lasted for 30–60 s, alternating with a 30–60 s rest break, in the ratio of exercise time to rest 1:2, 1:1 or 2:1, according to the individual capabilities of the participant and considering the training progression and stage of pregnancy. After the interval part of the training, participants performed resistance, postural, neuromotor (e.g., body balance) and stretching exercises which lasted approx. 5–10 min. The cool down part consisted of pelvic floor muscle exercises and preparation-for-birth exercises, e.g., birth position and breathing exercises (5–10 min) as well as relaxation and visualization of pregnancy and childbirth (5–15 min). Women did not need any equipment during exercises and only resistance of own body was applied. Women were able to participate in the training program regardless of their level of fitness or exercise capacity, as well as the level of motor skills (based on the diagnostic exercise tests, the exercise program was tailored to the individual needs and capabilities of a woman (50, 51)).

Group HIIT sessions were provided online, from 9.30 to 10.30 a.m. with the use of the MS Teams^®^ platform on Mondays, Wednesdays and Fridays, except one Monday which was a holiday (in total there were 23 sessions). Women took part in 19 ± 4 sessions on average (80% of the entire HIIT exercise program). Prior starting the program, participating women were trained on how to use the MS Teams^®^ application, as well as they were informed about the safety rules for exercising at home (including the safe organization of space at home, rules of communication in the event of an accident or deterioration of well-being). The whole HIIT program was supplemented by educational class once a week. All sessions were conducted by the principal researcher, who is a graduated fitness professional and certified Pregnancy and Postnatal Exercise Specialist according to the European educational standard for this profession (52). Email and phone contact were used in order to monitor the adherence to the program.

The control group (EDU group) consisted of 16 pregnant women who participated in educational sessions on a healthy lifestyle, physical activity in the perinatal period and selected aspects of pregnancy and motherhood (this was the same educational program as for the HIIT group). Educational classes were provided online in real time, 1 h once a week for 8 weeks. Women from the EDU group were encouraged to individually undertake exercise and fulfill at least the recommended level of physical activity (minimum 150 min per week of moderate to vigorous intensity). They were asked to keep a diary of all their physical activity (including both structured exercise sessions and daily activities lasting at least 10 min, such as cleaning the house, gardening, shopping). Of note, the EDU group did not monitor the intensity with heart rate monitors, but used the RPE scale and Talk Test. We recommended exercise intensity at a level in which women felt a marked increase in breathing frequency, but until their breathing interfered with their conversation. On average, the participants reported 20 bouts of physical activity with an average intensity of 6 ± 0.6 on the 0–10 RPE scale.

During the entire study, all participating women remained under standard obstetric care. Both HIIT as well as EDU interventions were not associated with any negative effects on the course of pregnancy or on childbirth parameters. We collected data on obstetric and neonatal postpartum outcomes, using an online questionnaire (based on medical records).

**Figure 2:**
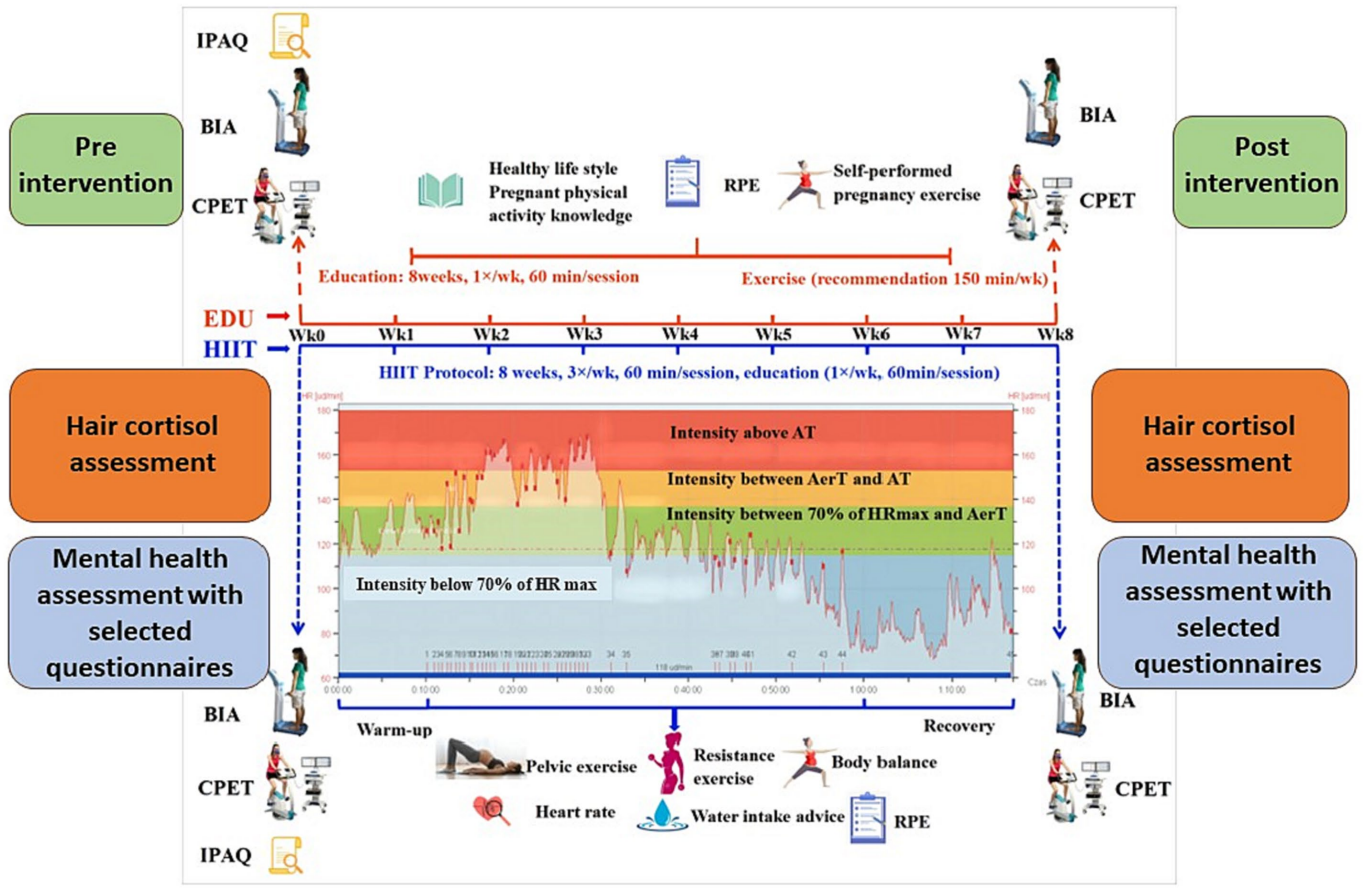
Schematic representation of the study design. AerT, aerobic threshold; AT, anaerobic threshold; BIA, bioelectrical impedance analysis; CPET, cardiopulmonary exercise test; EDU, education; HIIT, high-intensity interval training; HR_max_, maximal heart rate; IPAQ, International Physical Activity Questionnaire; RPE, rating of perceived exertion. Figure modified based on the Figure 2 published by Yu et al. (53).

We used the G∗power version 3.1.3. software in order to predetermine sample size (on the basis of the power calculation). The minimal sample size of 32 (16 for each group) with an allocation ratio 1:1, a power of 0.9 and alpha of 0.05 was predetermined on the basis of the values of the mean and SD from preliminary tests with 8 women form the HIIT group and 8 women from the EDU group. The study design is presented on the Figure 2.

The study was conducted in the Laboratory of Physical Effort and Genetics in Sport, at Gdansk University of Physical Education and Sport in Gdansk, Poland, in 2021. This research was performed according to the principles of the WMA Declaration of Helsinki and with the approval of the Bioethics Commission at the District Medical Chamber in Gdansk (KB-8/21). All participants were asked to sign the informed consent prior testing. The study protocol was registered in ClinicalTrials.gov (NCT05009433). After trial commencement no significant methodological changes were introduced. We have followed standards for transparency, openness and reproducibility of research and also adhered to the CONSORT standards (54, 55). No data manipulations were performed. Outcomes of this study are available by emailing the corresponding author. The data analysis presented in this work was not preregistered.

### Statistical analysis

Statistical tests were performed using the IBM Statistical Package for the Social Sciences version 27.0 (IBM Corp., Armonk, New York, United States), with the statistical significance set to *p* < 0.05. The analysis of the normality of the distribution of study variables was developed using the Kolmogorov–Smirnov test (K–S test). Inter-and intra-group mean differences were analyzed by the Student’s t-test or analysis of variance (ANOVA) test when appropriate. In the case of distributions which were significantly different from the normal distribution, we used the non-parametric Friedman test, Mann–Whitney U test and the Wilcoxon T test for the assessment of inter-and intra-group differences, respectively. Additionally, Chi-square was used to evaluate the differences in frequencies. We also conducted Intention-to-treat (ITT) analyses using linear interpolation to estimate the results of participants who were lost during the study. The total group analyzed for the purpose of ITT analysis.

## Results

### Characteristics of the study participants

The characteristics of women participating in our study are presented in Table 1. Groups did not differ in terms of age, BMI, initial VO_2max_, initial weekly PA, depressive symptoms, physical health, and fear of childbirth. As the endocrine system undergoes various changes during pregnancy, importantly the HIIT group and the EDU group were at similar gestational weeks (M = 20.68, SD = 4.21, and M = 23.37, SD = 4.03, respectively; *p* = 0.055). However they differed significantly in mental health indicator. The baseline levels of hair cortisol also differed significantly between groups (see the details in Table 1). Nevertheless, in both groups the cortisol levels were within the values typical for the second trimester of pregnancy (between 0 and 34.15 pg./mg), in accordance with meta-analysis by Marceau et al. (25). Therefore, we assumed the differences did not have clinical significance.

**Table 1:** The characteristics of the study participants.

Variable	Group				
	HIIT *n* = 22, M ± SD	EDU *n* = 16, M ± SD	Statistics^1^	Value of *p*	Cohen’s d
Age (years)	30.18 ± 4.21	32.38 ± 3.52	*Z* = −1.932	0.053	0.656
BMI (height/weight^2^)	23.78 ± 2.64	24.85 ± 3.14	*t* = −1.143	0.261	0.376
Week of gestation	20.68 ± 4.21	23.37 ± 4.03	*t* = −1.981	0.055	0.651
Initial VO_2max_ (kg/ml/min)	25.38 ± 4.14	24.14 ± 3.43	*Z* = −0.972	0.331	0.458
Initial weekly PA (METs)	2833.27 ± 2196.31	2719.38 ± 2967.41	*Z* = −0.636	0.525	0.207
Hair cortisol (pg/mg)	**13.26 ± 18.10**	**17.80 ± 9.92**	***Z* = −2.218**	**0.027**	**0.771**
Depressive symptoms (BDI-II score)	5.68 ± 4.81	4.56 ± 2.63	*t* = 0.842	0.405	. 277
Physical health (PCS score)	47.23 ± 6.73	46.64 ± 5.50	*t* = 0.287	0.776	0.094
Mental health (MCS score)	**48.55 ± 7.67**	**54.18 ± 4.90**	***t* = −2.575**	**0.014**	**0.846**
Fear of childbirth (CAQ score)	32.05 ± 5.75	34.44 ± 3.95	*t* = −1.433	0.160	0.471

^1^In case of variables with the distribution close to normal distribution we used parametric testing with Student t test and in case of variables with a distribution significantly different from the normal distribution we used non-parametric testing with Mann–Whitney *U* test. Bold type indicates significant difference in the outcome variable.

### Hair cortisol

The scores obtained for the hair cortisol were significantly different from the normal distribution in case of the scores for HIIT group in the Time 1 assessment (*p* < 0.05), and non-significant in Time 2 assessment in the HIIT group and in both measurements in the EDU group (*p* > 0.05). Thus, in case of the between-group differences in Time 1 we used Mann–Whitney U test and parametric Student’s t-test in Time 2 assessment. We found significant between-group difference in the Time 1 (*Z* = −2.218, *p* = 0.027, Cohen’s *d* = 0.771), as well as Time 2 assessments (*t* = 2.514, *p* = 0.017, Cohen’s *d* = 0.826). The results are presented in Table 2 and Figure 2.

**Table 2:** The changes in the hair cortisol levels and psychological variables before and after 8-week intervention in the HIIT (*n* = 22) and EDU (*n* = 16) groups.

	Time 1					Time 2			
Variable			Between-group differences				Between-group differences		
	Group		Statistics	Value of *p*	Cohen’s d		Statistics	Value of *p*	Cohen’s d
Hair cortisol (pg/mg)	HIIT	**13.26 ± 18.10**	**Z = −2.218**	**0.027**	**0.771**	**17.2 ± 8.36**	**t = 2.514**	**0.017**	**0.826**
	EDU	**17.80 ± 9.92**				**10.7 ± 6.90**			
Depressive symptoms (BDI score)	HIIT	5.68 ± 4.81	*t* = 0.842	0.405	0.277	4.95 ± 3.70	Z = −0.716	0.474	0.232
	EDU	4.56 ± 2.63				4.06 ± 1.95			
Physical health (PCS score)	HIIT	47.23 ± 6.73	t = 0.287	0.776	0.094	8.10 ± 1.73	t = 0.094	0.925	0.031
	EDU	46.64 ± 5.50				6.53 ± 1.63			
Mental health (MCS score)	HIIT	**48.55 ± 7.67**	**t = −2.575**	**0.014**	**0.846**	51.92 ± 6.56	t = −1.079	0.288	0.355
	EDU	**54.18 ± 4.90**				54.00 ± 4.73			
Fear of childbirth (CAQ score)	HIIT	32.05 ± 5.75	t = −1.433	0.160	0.471	31.14 ± 7.47	t = 0.408	0.686	0.134
	EDU	34.44 ± 3.95				30.13 ± 7.66			
Initial VO_2_max (kg/ml/min)	HIIT	25.38 ± 4.14	*Z* = −0.972	0.331	0.328	**24.91 ± 5.13**	***t* = 2.785**	**0.008**	**0.915**
	EDU	24.14 ± 3.43				**20.60 ± 4.03**			
Initial weekly PA (METs)	HIIT	2833.27 ± 2196.31	*Z* = −0.636	0.525	0.207	3038.02 ± 2232.35	*Z* = −1.065	0.287	0.351
	EDU	2719.38 ± 2967.41				2733.59 ± 3733.37			

HIIT group consisted of 22 women; EDU group consisted of 16 women. Bold type indicates significant difference in the outcome variable. ^2^In case of variables with the distribution close to normal distribution we used parametric testing with Student t test and in case of variables with a distribution significantly different from the normal distribution we used non-parametric testing with Mann–Whitney *U* test.

We used Wilcoxon’s test to verify the within group differences in the cortisol levels in the HIIT group and Student’s t test for dependent variables in the EDU group. We found significant differences between Time 1 and Time 2 assessments both in the HIIT group (*Z* = −1.964, *p* = 0.05) as well as in the EDU group (*t* = 2.017, *p* = 0.044). An important result of this analysis is that cortisol levels increased significantly in the HIIT group and decreased in the EDU group.

Since both groups differed significantly in the first assessment, we decided to add additional information about the relationship between the initial scores for hair cortisol and the magnitude of change in both groups. To do so we decided to correlate the values obtained in the first assessment and the values of the difference between the two assessments (*d* = T2-T1) for both groups. With this additional information we were able to indicate that the increase of cortisol between two assessments was greater among women with initial lower cortisol in both groups (correlation strong and significant in HIIT group: Pearson’s *r* = −0.9 and EDU group: Pearson’s *r* = −0.85).

Worth to mention is also the fact that in the HIIT group the increase in hair cortisol was observed among 72.73% of participants, and in the EDU group among 18.75%. The difference between these two groups was statistically significant (*Pearson Chi-Square* = 10.795, *p* = 0.001).

**Figure 3:**
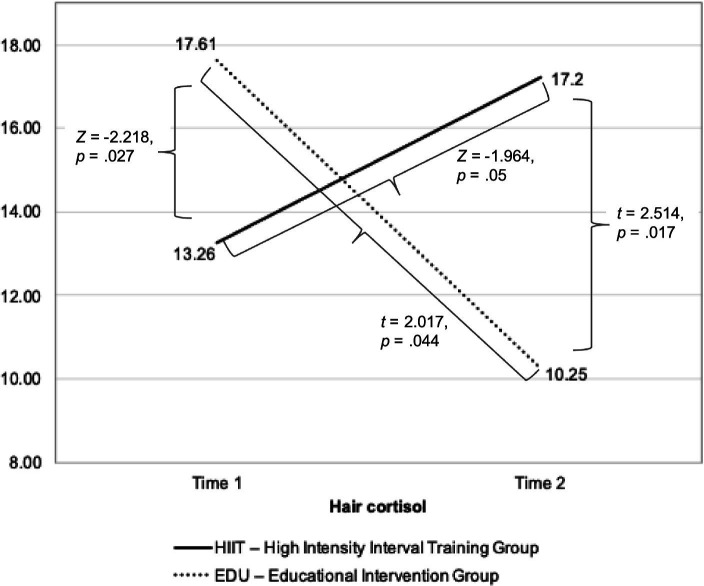
The changes in the hair cortisol levels before and after 8-week intervention in the HIIT and EDU groups.

The Intention-to-treat analysis was conducted on the scores coming from all 66 participants with the use of linear interpolation. The analysis revealed similar results as the above-described analysis: that both groups differed significantly in both—Time 1 and Time 2 assessments. However, although in the EDU group we found significant difference between the two assessments, no change in this aspect was found in the HIIT group (Figure 3).

### Depression symptoms

We have started our analyses from the Kolmogorov–Smirnov test to verify the distributions of the variables. The analysis revealed that in case of the BDI score the distributions of the variables were close to the normal distribution in Time 1 and Time 2 in the EDU group and Time 1 in the HIIT group (*p* > 0.05) and significantly different from normal distribution in Time 2 in the HIIT group. Groups did not differ in depressive symptoms both in the first measurement (*t* = 0.842, *p* = 0.405, Cohen’s *d* = 0.277) as well as in the second measurement (*Z* = −0.716, *p* =. 474, Cohen’s *d* = 0.232). The within group differences between first and second assessment were insignificant both in the HIIT group (*Z* = −1.116, *p* = 0.265, Cohen’s *d* = 0.490) as well as in the EDU group (*t* = 0.612, *p* = 0.549, Cohen’s *d* = 0.153). See Table 2 for the details.

The ITT analysis confirmed that the difference between the two groups was statistically non-significant in both—the Time 1 and Time 2 assessments. However, contrary to the above-described outcomes the differences between Time 1 and Time 2 were statistically significant in both – the HIIT (*Z* = −3.206, *p* = 0.001, Cohen’s *d* = 1.29; M_initail_ = 8.91, SD = 6.54, M_final_ = 4.91, SD = 3.83), and the EDU group (*t* = 5.390, *p* < 0.001, Cohen’s *d* = 0.968; M_initail_ = 8.92, SD = 5.05, M_final_ = 3.60, SD = 1.78).

Of note, as sleep is one of the factors that significantly influences cortisol concentrations, we additionally decided to verify whether there were any between-group differences in sleep patterns. In order to do so, we verified the differences between the two groups in answers on item 16 in *BDI-II* (were women indicated how well they sleep). In the Time 1 assessment, in the HIIT group half of participants (50%) indicated that they slept as well as before, 45.45% indicated they slept worse than before, and 1 woman indicated that she woke up 1–2 h early and had a hard time getting back to sleep. In the EDU group 43.75% of women pointed that they slept as well as before, the same percentage of women indicated that they slept worse than before, 1 woman indicated that she woke up 1–2 h early and had a hard time getting back to sleep, and 1 woman pointed that she woke up a few hours early and could not fall asleep. We found no significant between-group differences in those sleep patterns in the Time 1 assessment (*Chi-Square* = 1.509, *p* = 0.680). Importantly, the groups also did not differ substantially in the Time 2 measurement (*Chi-Square* = 0.567, *p* = 0.904). Due to the specificity of the data (measured on a rank scale, with a large percentage of the same outcomes), we did not perform analyses in this study to correlate sleep quality with the level of hair cortisol.”

### Physical and mental health

The scores obtained for the physical health indicator in the SF-12 assessment were close to the normal distribution in the Kolmogorov–Smirnov test (*p* > 0.05). ANOVA with repeated measures revealed that both the main effect (*F* = 0.055, *p* = 0.816, *η^2^* = 0.002, observed power = 0.056) as well as interaction effect (*F* = 0.026, *p* = 0.873, *η^2^* = 0.001, observed power = 0.053) were statistically insignificant. Post-hoc analyses revealed that between group differences were insignificant both in the first assessment (*t* = 0.287, *p* = 0.776, Cohen’s *d* = 0.094) as well as in the second measurement (*t* = 0.094, *p* = 0.925, Cohen’s *d* = 0.031). The differences between first and second measurements were insignificant both in the HIIT group (*t* = 0.257, *p* = 0.800, Cohen’s *d* = 0.055) as well as in the EDU group (*t* = 0.074, *p* = 0.942, Cohen’s *d* = 0.018). The details are presented in Table 2.

The ITT analysis confirmed all above-described outcomes.

In case of the mental health indicator in the SF-12 assessment the scores were not significantly different from the normal distribution (*p* > 0.05). ANOVA with repeated measures revealed that both the main effect (*F* = 2.228, *p* = 0.144, η^2^ = 0.058, observed power = 0.306) as well as interaction effect (*F* = 2.784, *p* = 0.104, η^2^ = 0.072, observed power = 0.369) were statistically insignificant. Post-hoc analyses revealed that between group differences were significant in the first assessment (*t* = −2.575, *p* = 0.014, Cohen’s *d* = −0.846) however in the second measurement the differences were statistically insignificant (*t* = −1.079, *p* = 0.288, Cohen’s *d* = −0.355). The differences between first and second measurements were significant in the HIIT group (*t* = −2.125, *p* = 0.046, Cohen’s *d* = −0.453) and insignificant in the EDU group (*t* = 0.154, *p* = 0.879, Cohen’s *d* = 0.039). The details are presented in Table 2.

Here, similarly as in case of cortisol level, both groups differed significantly in the first assessment, thus we decided to add additional information about the relationship between the initial scores for mental health and the magnitude of change in both groups. We did similar analysis as previously for cortisol: correlation of the values obtained in the first assessment and the values of the difference between the two assessments (*d* = T2-T1) for both groups. With this additional information we were able to indicate that the increase of mental health indicator between two assessments was greater among women with initial lower result in both groups, however the correlation was weaker (but still significant) than in case of the cortisol level (in HIIT group: Pearson’s *r* = −0.62 and in EDU group: Pearson’s *r* = −0.53).

In the HIIT group the increase in mental health score was observed among 64.64% of participants, and in the EDU group among 37.50%. However, the difference between these two groups was statistically insignificant (*Pearson Chi-Square* = 2.538, *p* = 0.111).

The ITT analysis revealed that contrary to the above-described outcomes groups did not differ significantly in mental health during the Time 1 assessment (*Z* = −1.741, *p* = 0.082, Cohen’s *d* = 0.439), however we found significant difference in the Time 2 assessment (*Z* = −2.056, *p* = 0.040, Cohen’s *d* = 0.523). In HIIT group the difference between Time 1 and Time 2 assessments was insignificant (*Z* = −1.114, *p* = 0.265, Cohen’s *d = 0*.277; M_initail_ = 49.59, SD = 9.42, M_final_ = 51.91, SD = 5.90). In the EDU group the difference between Time 1 and Time 2 was statistically significant (*Z* = −3.645, *p* < 0.001, Cohen’s *d =* 1.004; M_initail_ = 46.02, SD = 9.33, M_final_ = 54.83, SD = 3.93).

### Fear of childbirth

In case of the fear of birth the scores were not significantly different from the normal distribution (*p* > 0.05). ANOVA with repeated measures revealed that both the main effect (*F* = 3.564, *p* = 0.067, η^2^ = 0.090, observed power = 0.451) as well as interaction effect (*F* = 1.514, *p* = 0.227, η^2^ = 0.040, observed power = 0.224) were statistically insignificant. Post-hoc analyses revealed that between group differences were insignificant both in the first assessment (*t* = −1.433, *p* = 0.160, Cohen’s *d* = −0.471) as well as in the second measurement (*t* = 0.408, *p* = 0.686, Cohen’s d = 0.134). The differences between first and second measurements were significant in the EDU group (*t* = 2.444, *p* = 0.027, Cohen’s *d* = 0.611) and insignificant in the HIIT group (*t* = 0.460, *p* = 0.650, Cohen’s *d* = 0.098). The details are presented in Table 2. The ITT analysis confirmed the above-described outcomes.

### Level of physical activity and VO_2max_

We used the Kolmogorov–Smirnov test to verify the distributions of the level of physical activity and the VO_2max_ variables. The analyses revealed that in case of level of physical activity the distributions of the variables were significantly different from the normal distribution in Time 2 in the HIIT group and in Time 1 and Time 2 in the EDU group (*p* < 0.05). Thus, we decided to use the Mann–Whitney U statistics to verify the inter-group differences and Wilcoxon’s test to verify the within-group differences. The between-group differences were statistically insignificant both in Time 1 assessment (*Z* = −0.636, *p* = 0.525, Cohen’s *d* = 0.207) as well as Time 2 assessment (*Z* = −1.065, *p* = 0.287, Cohen’s *d* = 0.351). The within group differences were non-significant both in the HIIT group (Z = −0.568, *p* = 0.570, Cohen’s *d* = 0.185) as well as EDU group (*Z* = −0.103, *p* = 0.918, Cohen’s *d* = 0.033). See Table 2 for the details. The ITT analysis confirmed the above-described outcomes.

The analyses revealed that in case of level of VO_2max_ the distributions of the variables were significantly different from the normal distribution in Time 1 in the HIIT group (*p* < 0.05). All other distributions were close to the normal distribution (*p* > 0.05). Thus, we decided to use the Mann–Whitney U statistics to verify the inter-group differences in Time 1 and Student’s t-test in Time 2 assessment. To verify the within-group differences we used Wilcoxon’s test in the HIIT group and Student t-test for dependent variables in the EDU group. The between-group differences were statistically insignificant in Time 1 assessment (*Z* = −0.972, *p* = 0.331, Cohen’s *d* = 0.328) however in Time 2 assessment we found significant between-group difference (*t* = 2.785, *p* = 0.008, Cohen’s *d* = 0.915). The within group differences were non-significant in the HIIT group (Z = −0.262, *p* = 0.794, Cohen’s *d* = 0.087) however in case of the EDU group the difference was statistically significant (*t* = 4.903, *p* < 0.001, Cohen’s *d* = 1.226). See Table 2 for the details. The ITT analysis confirmed the above-described outcomes.

### Correlation analyses between hair cortisol levels and other variables

In the next step of the analyses we decided to analyze the correlation between hair cortisol in both assessments with the depression symptoms, physical and mental health, fear of childbirth, level of physical activity and VO_2max_ in both measurements. We have found no significant correlations between these variables both in the HIIT group as well as in the EDU group. The results are presented in Table 3.

**Table 3:** Correlations (Pearson’s r) between hair cortisol levels and the severity of depression symptoms, physical and mental health, fear of childbirth, level of physical activity and VO_2max_ in both groups in the two assessments.

Variable	HIIT		EDU	
	Cortisol in hair in Time 1 (Pearson’s r coefficient)	Cortisol in hair in Time 2 (Pearson’s r coefficient)	Cortisol in hair in Time 1 (Pearson’s r coefficient)	Cortisol in hair in Time 2 (Pearson’s r coefficient)
Time 1: depression symptoms	−0.054	0.083	0.313	−0.204
Time 1: physical health	0.051	−0.154	−0.102	0.022
Time 1: mental health	−0.170	0.075	0.071	−0.065
Time 1: fear of childbirth	0.313	0.127	−0.130	0.040
Time 1: level of physical activity	−0.215	0.074	0.032	−0.208
Time 1: VO_2_max	−0.102	−0.189	0.425	−0.226
Time 2: depression symptoms	−0.231	−0.266	0.079	−0.200
Time 2: physical health	0.018	0.164	−0.285	0.172
Time 2: mental health	0.130	−0.018	−0.229	0.171
Time 2: fear of childbirth	−0.411	−0.126	0.436	−0.115
Time 2: level of physical activity	−0.018	−0.043	0.040	0.057
Time 2: VO_2_max	−0.021	−0.041	0.317	−0.090

*Correlation significant at *p* < 0.05 (two-sided). **Correlation significant at *p* < 0.01 (two-sided).

We also did not found any significant correlations between the post-pre intervention change in hair cortisol (Cortisol in hair in Time 2—Cortisol in hair in Time 1) and the post-pre intervention change (variable in Time 2—variable in Time 1) in the depression symptoms, physical and mental health, fear of childbirth, level of physical activity and VO_2max_. See Table 4 for the details.

**Table 4:** Correlations (Pearson’s r) between post-pre intervention change in hair cortisol and the post-pre-intervention change in the depression symptoms, physical and mental health, fear of childbirth, level of physical activity and VO_2max_.

Variable	Change in hair cortisol	
	HIIT	EDU
Change in depression symptoms	−0.015	0.181
Change in physical health	0.153	0.342
Change in mental health	−0.316	0.349
Change in fear of childbirth	0.420	−0.496
Change in level of physical activity	0.018	−0.109
Change in VO_2_max	−0.166	0.124

*Correlation significant at *p* < 0.05 (two-sided). **Correlation significant at *p* < 0.01 (two-sided).

## Discussion

This paper aimed to fill the knowledge gap by exploring the relationship between hair cortisol levels, mental health outcomes and the effects of HIIT in pregnant women. By investigating the potential mechanisms underlying the protective effects of HIIT on mental well-being during pregnancy, we aim to provide evidence-based insights that can inform prenatal care strategies and improve the overall health and well-being of expectant mothers and their offspring. The most important finding of our study is that women attending HIIT intervention responded in a different direction in the production of cortisol compared to the EDU group performing standard moderate intensity physical activity. In the HIIT we observed the increase in the hair cortisol level (72.73% of women), and in the EDU group there was a substantial decrease (81.25%). The ITT analysis confirmed our findings. Thus, HIIT stimulated the production of cortisol, but it did not have a negative effect on the analyzed variables related to fear of childbirth and psychophysical condition during pregnancy. On the contrary, we observed a significant improvement in mental health only in the HIIT group. While cortisol is often referred to as a stress hormone, HIIT has been found to have a complex relationship with cortisol levels. One possible explanation for the increase in cortisol levels after HIIT among pregnant women is the physiological stress response triggered by the high-intensity nature of the exercise. HIIT involves pushing the body to its limits and can be physically demanding, which may activate the body’s stress response system and lead to cortisol release. Some studies suggest that acute bouts of HIIT can temporarily increase cortisol levels immediately after exercise (56, 57). Of note, in our study increase of cortisol was greater among those women with initial lower cortisol and smaller among those with initial higher cortisol level. This observation is also a justification to treat HIIT as an activity that stimulates a positive stress response, which may activate the body’s adaptive mechanisms, e.g., strengthening tissues and cells that are subjected to more difficult conditions. At the same time, it does not pose a risk of increasing stress, which could have a harmful effect (as it does not cause a substantial increase in those who already have a “baseline” higher level of stress). This so called ‘hormesis hypothesis’ assumes that a stressor that is harmful to the body in larger doses can have a positive—mobilizing—effect when it is moderate (58, 59). Therefore, our research illustrates this effect: moderate physical activity mobilizes the body (mainly in those whose body was initially poorly activated), which is reflected in an increase in cortisol, but within normal limits without harmful effects. In the group of women subjected to educational interventions, an increase in cortisol occurred in a small percentage of women. We observed a decrease in most of participants in this group, which may be due to the fact that as pregnancy progresses, women tend to avoid high intensity exercise and thus the adaptive mechanisms are not stimulated enough (which may have negative health effects) at. However, this last hypothesis requires confirmation in further research.

A study conducted by Jurgelaitiene et al. (13), explored the effects of stress and fatigue after intense exercise. Contrary to previous assumptions that these effects are localized solely in the muscles, the study suggests that the central nervous system (CNS) also plays a critical role in influencing emotional regulation and mood, which are associated with various mental conditions. This finding helps to explain our own observations that high-intensity interval training (HIIT) as well as educational (EDU) intervention, positively impacted anxiety states and depressive symptoms in pregnant women. The meta-analysis conducted by Niven et al. (60), further emphasizes that individuals tend to have a less positive experience during high-intensity interval training. However, they report that post-exercise, it is perceived as more enjoyable. Furthermore, in the results of the current study, HIIT was found to have better training effects on exercise capacity parameters compared to self-performed physical activity practiced by EDU group. It demonstrates that the repetition of HIIT sessions led to a high level of physical activity and maintained a stable VO_2max_, providing valuable insights into the pregnant woman’s ability to meet the increased oxygen demands during exercise and daily activities (61). Additionally, it sheds light on the potential impact of HIIT on pregnant women’s stamina.

This all suggest that pregnant women who participated in HIIT benefited from stress which is in accordance with current psychological models emphasizing that the stress response can be differentiated into both negative-distress and positive aspects-eustress. In the past two decades, there has been a growing interest in exploring the positive aspects of stress, coinciding with the emergence of positive psychology. This approach shifts the traditionally deficit-oriented focus of stress research toward highlighting positive human resources (62). Additionally, the results of our study challenge the notion that cortisol levels should always be considered a marker of depression or anxiety during pregnancy, contrary to recommendations made by other authors (26, 63). It suggests that cortisol may not be a reliable indicator in all cases and should be interpreted with caution when assessing mental health during pregnancy. It is also worth emphasizing that in the analysis of the current study, no correlations were found between hair cortisol and psychological variables such as depression symptoms, fear of childbirth, and mental health. In summary, while some studies suggest that HIIT may increase cortisol levels among pregnant women, the significance and potential implications of this increase are still being explored as long as the values of cortisol levels remain inconclusive (25). It is important to note that exercise-induced cortisol responses in the participants of our study are generally considered normal and not necessarily detrimental to health (56, 57). Further research is needed to better understand the relationship between HIIT, cortisol response, and the potential short-term and long-term effects on maternal and fetal health.

The fear of childbirth and anxiety surrounding the labor and delivery process are widespread concerns among pregnant women, significantly influencing their overall well-being and birthing experience (64, 65). Consequently, a remarkable and notable discovery from our study emerged: the HIIT group experienced statistically significant enhancement in their mental health. While the outcome in the ITT analysis was not statistically significant, the trend was still beneficial. This finding highlights the exceptional potential of high-intensity interval training in addressing and alleviating these concerns, showcasing its substantial positive impact on the emotional well-being of expectant mothers. It is important to note that both the HIIT and EDU groups had initially low levels of depressive symptoms. In addition to our findings, the EDU group experienced a significant decrease in the fear of childbirth. Similarly, the fear of childbirth decreased in the HIIT group, but the difference was statistically insignificant. Nevertheless, it is truly inspiring to see that engaging in the HIIT program potentially led to a reduction in childbirth fear, indicating a positive impact on the participants’ mindset. This suggests that regular physical activity routines could have a valuable and beneficial effect on overall childbirth experiences among pregnant women. The previous studies also confirm these observations that exercise, through improving physical fitness and stamina, could be a suitable intervention to alleviate fear of childbirth, as it may enhance the woman’s confidence in her physical capabilities and foster a sense of empowerment (8–10, 66–68). Therefore, HIIT, with its time-efficient nature and potential for enjoyment, may provide similar mental health benefits as other exercise modalities.

To better understand the influence of intense training on women’s mental and physical health, it is important to acknowledge the limitations of this study and take necessary steps. Moreover, this study builds upon our previous work, where our focus was on examining alterations in psychological variables among a broader cohort of pregnant individuals who underwent HIIT (8). Despite the smaller number of pregnant subjects in the current study due to the method of measuring cortisol (hair sample could not be dyed), we decided to continue the analysis because the results revealed appeared interesting and there is a big gap in the literature on this topic. It is important to note that despite the initial differences in baseline cortisol levels between the HIIT and EDU groups, these variances do not hold clinical significance (25). The recruitment criteria for this study, in fact, revolved around the week of pregnancy, and most other factors such as psychological variables, which generally did not display divergence among subjects at baseline. Consequently, it was plausible to assume that both groups might exhibit similar responses to the intervention since they were similar in terms of their initial psychological measurements.

Worth exploring in further studies is the association between cortisol levels in women who trained HIIT during pregnancy and their psychological functioning postpartum. The question which researchers can try to answer in their future studies is whether increase in cortisol levels in HIIT participants during pregnancy can be recognized as ‘eustress’ type response that boost mental health of women postpartum. Another area worth further exploration is the relationship between cortisol levels and psychological variables among postpartum women who continue to participate in HIIT. It is worth to explore in a such approach whether cortisol levels stabilize or change in comparison to the levels during pregnancy and how this change relates to changes in psychological variables. Due to the fact that sleep pattern is one of the factors that significantly influences cortisol concentrations, in the future studies it is also worth to include specific tools to measure the amount and quality of sleep. Especially since sleep quality usually deteriorates as pregnancy progresses.

## Conclusion

The HIIT program in pregnancy, compared to the standard program of moderate-intensity physical activity, affects the production of cortisol in a different way. In the HIIT group we observed the increase in the hair cortisol levels, and in the EDU group there was a substantial decrease of this hormone. Nevertheless, the increase in cortisol in the HIIT group was not related with negative outcomes. We found no associations between hair cortisol levels and the severity of depressive symptoms, psychophysical well-being or fear of childbirth. Therefore, in the light of our research, the use of cortisol levels in pregnancy as a marker of negative stress, including the risk of depression, seems to be unjustified. The HIIT programs should be promoted in pregnant women as safe, beneficial and healthy.

## Data availability statement

The raw data supporting the conclusions of this article will be made available by the authors, without undue reservation.

## Ethics statement

The studies involving humans were approved by Bioethics Committee for Scientific Research—Medical University of Gdansk. The studies were conducted in accordance with the local legislation and institutional requirements. Written informed consent for participation in this study was provided by the participants’ legal guardians/next of kin. Written informed consent was obtained from the individual(s) for the publication of any potentially identifiable images or data included in this article.

## Author contributions

DW: Conceptualization, Investigation, Methodology, Visualization, Writing – original draft, Writing – review & editing. TW-K: Formal analysis, Methodology, Software, Validation, Visualization, Writing – original draft, Writing – review & editing. RS-R: Writing – review & editing. RL: Methodology, Writing – original draft. AS: Conceptualization, Data curation, Funding acquisition, Investigation, Methodology, Project administration, Resources, Supervision, Visualization, Writing – original draft, Writing – review & editing.
